# Encouraging the Voluntary Mobilization of Mental Resources by Manipulating Task Design: Explorative Study

**DOI:** 10.2196/63491

**Published:** 2025-04-10

**Authors:** Lina-Estelle Louis, Saïd Moussaoui, Sébastien Ravoux, Isabelle Milleville-Pennel

**Affiliations:** 1 École Centrale Nantes, Centre National de la Recherche Scientifique (CNRS), Laboratoire des Sciences du Numérique de Nantes (LS2N), UMR 6004, Nantes Université, Nantes, 44321, France, +33 240376918; 2 Entreprise Onepoint, Nantes, France

**Keywords:** visual game-like elements, gamification, multiplicity of cognitive functions, cognitive tasks, perceived playfulness, mental workload, performance, cognitive training, aging, mental effort, cognitive function, cognitive skills, cognitive tests

## Abstract

**Background:**

Cognitive training is increasingly being considered and proposed as a solution for several pathologies, particularly those associated with aging. However, trainees need to be willing to invest enough mental effort to succeed and make progress.

**Objective:**

In this study, we explore how gamification in a narrative context (ie, the addition of visual game-like elements [GLEs] embedded in real-world contexts) could contribute increase in perceived playfulness (PP) and voluntary mental effort allocated to a cognitive task. In such context, narrative elements and GLEs can be designed to align with a commonly relatable scenario (like simulating fishing or gardening activity) to ground the task in familiar, real-world contexts. We also consider if the supposed effect of GLEs on PP and voluntary mental effort could endure while manipulating an intrinsic variable of the task (ie, by increasing cognitive solicitation).

**Methods:**

In total, 20 participants (average age 33.6, SD 8.6 y) took part in 3 cognitive tasks proposed in a numerical format: a classic version of the Corsi test (Classic Corsi, a spatial memory task), a playful version of the Classic Corsi test (Playful Corsi), with added visual GLEs in a narrative context, and a playful version of the Classic Corsi test with added cognitive solicitation, that is, mental motor inhibition (Playful Corsi Multi). We assessed the impact of visual GLEs and cognitive solicitation on PP (1 question) and mental workload (MWL) using NASA-Task Load Index (NASA-TLX) and workload profile (WP) questionnaires.

**Results:**

Results showed that PP was not influenced by interface’s playful characteristics (Classic Corsi [mean 62.4, SD 8.8] vs Playful Corsi [mean 66, SD 8.8]; W=77; *P*=.30) but decreased the time necessary to complete the task (Classic Corsi [mean 10.7, SD 2.1 s] vs Playful Corsi [mean 6.8, SD 1.6 s]; W=209; *P*<.001) as well as performance (Classic Corsi [mean 92.4, SD 9.1] vs Playful Corsi [mean 88.2, SD 11.3]; W=140.5; *P*=.02). So, possibly, visual GLEs could raise the stakes of the task slightly and implicitly encourage people to go a bit faster. Furthermore, visual GLEs increased MWL regarding attentional resources (assessed by WP: Classic Corsi [mean 52.4, SD 10.9] vs Playful Corsi [mean 65.8, SD 10.9]; W=27.5; *P*=.04), while manipulating cognitive solicitation impacted MWL when linked to task requirements (assessed by NASA-TLX: Playful Corsi [mean 54.2, SD 9.4] vs Playful Corsi Multi [mean 67.5, SD 9.4]; W=35.5; *P*=.01) without impacting the performance to the task (Playful Corsi [mean 83.8, SD 13.9] vs Playful Corsi Multi [mean 94, SD 5.5]; W=27; *P*=.007). Thus, working on the way cognitive functions are solicited would be wiser than adding visual GLEs to improve users’ voluntary mental effort while preserving performance.

**Conclusion:**

These results offer valuable insights to improve users’ experience during gamified cognitive tasks and serious games.

## Introduction

Cognitive training is increasingly being considered and proposed as a solution for several pathologies, particularly those associated with aging. However, trainees must be willing to invest enough mental effort to succeed and make progress. During the execution of an activity, we have the possibility of voluntarily activating our mental resources to reach a level of performance set by the task or by ourselves. This voluntary modulation of the quantity of mental resources invested when performing a task corresponds to the notion of mental effort [1-3]. Voluntary mental effort is a dimension of mental workload (MWL). The latter is a broader concept that also encompasses notions such as the perception of our own performance, or emotional states such as frustration. Voluntary mental effort should be distinguished from mental effort imposed by the task properties and undergone by the participant. Indeed, voluntary mental effort must be seen as a “positive” mental effort as it corresponds to a voluntary deployment of resources with positive consequences for the participant (high perceived performance and low frustration). Thus, in this context it should be considered regarding the corresponding perceived performance and frustration when performing the task. Positive mental effort should be accompanied by high perceived performance and low frustration. To make the notion of positive mental effort operational, we return to the definition of this concept, specifying that it is part of the MWL construct. In the scientific literature, NASA-Task Load Index (NASA-TLX) [2] and workload profile (WP) [4] questionnaires permit to measure MWL linked respectively to task requirements and the mobilization of attentional resources (since the WP employs the multiple resource theory of [5-7] as its foundation). NASA-TLX is particularly interesting for addressing the notion of positive mental effort, as it considers various dimensions of MWL, including mental effort, perceived performance and frustration, that we considered as crucial in the definition of positive mental effort.

Mental effort has been studied in various contexts since it is a key element for enhancing performance. However, few studies have focused on the notion of positive mental effort during cognitive training, which aims to enhance general cognitive skills such as memory, attention, or processing speed through repeated interventions using cognitive tasks or intellectually demanding activities [8-10]. Our aim is thus to study the factors that influence voluntary mobilization of mental effort when confronted to cognitive tasks used in a cognitive training process. To do that, we need to consider a task allowing to assess voluntary mobilization of mental effort but without the confounding effects associated with longitudinal cognitive training paradigms (reinforcement, error feedback, and repetition). Thus, in this study, our aim is not to design interventions for cognitive training, but rather to propose isolated cognitive tasks as controlled experimental tools to investigate the role of some factors. These factors could then be integrated in tasks developed for cognitive training. So, we wondered how we could encourage individuals to voluntarily mobilize greater mental effort to achieve better performance. To this end, we wanted to explore one possibility, that is, the gamification of a cognitive task.

Gamification is defined as the use of game-like elements (GLEs) in nongame contexts. GLEs are elements typically found in games such as visual elements (icons, colored patterns, evocative images, or playful animations), badges systems, narration, or leaderboards [9,11]. This definition of gamification differentiates it from serious games. Indeed, according to Vermeir et al [9], serious games use full-fledged games in nongame contexts, whereas gamification uses elements of a game integrated into real-world contexts (for instance, a cognitive task that uses colored patterns).

Furthermore, gamification can be linked to cognitive tasks, but its introduction may affect the validity of cognitive tests by potentially introducing new cognitive demands. Therefore, the gamification of cognitive tasks is most applied within the context of cognitive training [9,11-13]. The reason is because cognitive tasks are perceived as demotivating and frustrating given their repetitive and effortful aspects (Lumsden et al [14]) which may impact on perceived playfulness (PP). PP is the sense of being focused on an activity that is viewed as inherently enjoyable, fun, and interesting [15,16]. Higher PP results in higher user intention to perform a task [17], a stronger attitude toward using a particular technology [16], a better performance, and a higher effective response to computer training tasks [18]. Thus, we predict that the increasing of PP would impact the voluntary mobilization of mental resources and thus positive mental effort. There would therefore be a real interest in increasing the PP of a cognitive task during cognitive training. Integrating GLEs into cognitive tasks could be one solution, as their inclusion is supposed to increase PP [11,19].

Among all GLEs, performance feedback via a reward points system (the unit of measurement quantifying a user progression) is one of the most widely used [19] and therefore the most studied in the literature. Nevertheless, this system provides feedback on performance and thus contributes to its improvement. While it does indeed increase motivation and PP, it also introduces a bias in the sense that it induces a form of learning. In this study, we aimed to understand the factors that encourage the voluntary mobilization of mental resources in a cognitive task (positive mental effort), with the hypothesis that this mobilization will have a positive impact on performance. Introducing reinforcement would induce a sort of learning process that could mask the effect of other variables allowing to increase positive mental effort. Thus, we provide no reinforcement through performance feedback.

Consequently, only a selected few GLEs, specifically visual ones, which have no impact on performance feedback, should be incorporated. Visual GLEs can be defined as the deliberate arrangement of several visual variables, such as position and form [20], integrated into a particular context. Visual GLEs are contextualized with narrative elements (short story explaining the task and a visual surrounding corresponding to the story) to make them meaningful for the participant and add ecological aspect to the cognitive task. By ecological aspect, we mean a less arbitrary situation. The task has an objective that can be linked to a game that could be encountered in real life. For example, the narrative elements can be designed to align with commonly relatable scenarios, (like aiming at targets, arranging objects to solve a puzzle, navigating a maze, or simulating a fishing or gardening activity) to ground the task in familiar, real-world contexts. These elements serve two purposes, that is, (1) the short story provides a framework for participants to understand the task’s purpose, making the activity less abstract and (2) the visual surroundings (eg, buoys, and water effects) enhance immersion, reinforcing the narrative and fostering a sense of engagement. Together, these components aim to reduce the perceived arbitrariness of the cognitive task, encouraging participants to connect the task with a recognizable and meaningful activity from their own experiences.

Nevertheless, despite showing that GLEs increase PP [19], no study has specifically investigated the contribution of visual GLEs integrated with a narrative context within cognitive tasks and the impact on both PP and positive mental effort. Thus, our first aim (hypothesis 1) is to ascertain if visual GLEs integrated with a narrative context (which will be called a playful task) are enough to increase PP and then positive mental effort (characterized by higher perceived mental effort and performance but lower perceived frustration) when compared with a classic design. And therefore, we predicted that this increase in voluntary mental effort could also increase real performances.

Nevertheless, in everyday life, an individual must involve various amounts of cognitive functions to meet the requirements of an activity. The number of cognitive functions solicited is a variable intrinsic to task requirements which would have a direct impact on mental demands. Mental demands is the dimension of MWL directly related to the amount of mental activities required [2]. This could also impact mental effort imposed by the task itself. We can thus assume that this effect could also play a role in positive mental effort, notably via perceived performance and frustration. Thus, an increase in mental effort required by the task could lead to a decrease in positive mental effort. To our knowledge, no study has investigated the impact of increasing the solicitation of cognitive functions on PP, positive mental effort, or the resulting actual performances in a narrative ecological context. Thus, our second aim (hypothesis 2) is to determine if the multiplicity of cognitive solicitation of the playful task might decrease PP, positive mental effort (characterized by higher perceived effort and frustration but fewer perceived performance), and actual performances when compared with the simple playful task.

Hence, this study will explore how extrinsic features (visual GLEs within a narrative context) impact PP and positive mental effort, and whether this effect persists when manipulating intrinsic demands of the task (eg, the multiplicity of cognitive solicitation in a narrative context). To this end, we have set up a protocol containing various cognitive tasks. In the first step, we will consider the impact of adding GLEs to a standard cognitive task. Then we will investigate further the impact of adding cognitive solicitation to this task. The tasks and the logic behind their design are detailed in the Methods section.

## Methods

### Ethical Considerations

This study has received a positive decision from the Northwest III Ethics Commitee for the Protection of Persons, with the reference number n° 21.04612.000059, in accordance with the ethical guidelines for human participants research and the Declaration of Helsinki. Informed consent was obtained from all participants. All data were anonymized to ensure privacy and were securely stored on a protected cloud platform accessible only to the research team. No identifiable participant information is included in the manuscript or supplementary materials. At the end of the study, each participant received a €50 (US $54.69) gift voucher as compensation.

### Participants

In total, 20 (9 women, 11 men) took part in this experiment. Their average age was 33.6 (SD 8.6) years. All of them had at least a bachelor’s degree and 85% (17/20) of them had at least a bac+3 level. Furthermore, 70% (14/20) worked for Onepoint company, 20% (4/20) came from social network LinkedIn (Microsoft) and the others were acquaintances of the experimenters or from Nantes University Hospital.

### Recruitment

Participants were recruited by mailing an information letter inviting interested people to contact the experimenter if they wanted to participate in the study. At the reception of the mail, the experimenter contacted the participant in order to confirm (by oral statement) that he or she were healthy French-speaking volunteers residing in Nantes region that met the inclusion criteria (fluent in French, right-handed, having normal or corrected-to-normal vision, and having normal or corrected-to-normal hearing). Then the participant was informed about the experimental duration and procedure. After acceptance by the participant, an appointment was made for the experimental tests. The consent document was signed at the participant’s arrival, before the beginning of the experiment.

### Tasks and Procedure

#### Overview

The experimentation took place at Nantes University Hospital. Participants performed 3 experimental tasks in a random order on a screen positioned in front of them. All tasks were devised using Unity3D development software (version 2019.4.30f1; Unity Technologies). A link to the videos of all tasks is available in Multimedia Appendix 1.

To determine the level of complexity of the tasks, we considered several factors. Primarily, the complexity of a simple task (only soliciting 1 cognitive function) should not be arduous. Indeed, a simple task should accommodate the integration of an additional cognitive function in a condition that involves multiple cognitive functions. On the other hand, we did not want the simple task to appear too simplistic. Thus, we relied on the intermediate complexity levels from the study conducted by Louis et al [21], which offers a compromise considering the aforementioned conditions. Indeed, Louis et al [21] compared several cognitive tasks with different complexity levels, and the 5-item Corsi test induced an intermediate MWL (based on NASA-TLX) while still allowing high performance. Thus, the 5-item Corsi test is a sufficiently cognitively demanding task without being difficult. Consequently, to verify hypothesis 1 (visual GLEs integrated with a narrative context increase PP, positive mental effort, and performances) and 2 (the multiplicity of cognitive solicitation might decrease PP, positive mental effort, and performances), 3 digitized versions of the Corsi test were proposed, that are Classic Corsi [22], Playful Corsi, and Playful Corsi Multi.

#### Classic Corsi

**Figure 1.**
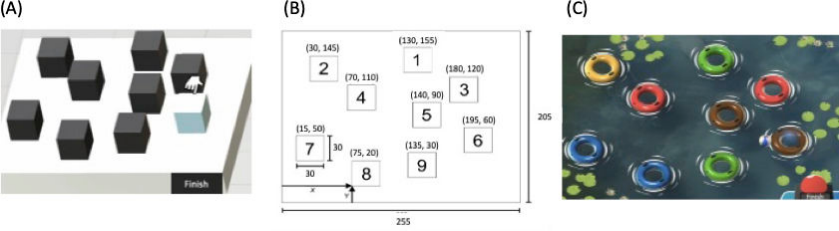
(A) Visual environment of the digitized version of the Classic Corsi test. (B) Positions of cubes in the Classic Corsi test from study by Kessels et al [23]. (C) Playful version of the digitized version of the Classic Corsi test: cubes are replaced by 9 buoys with different colors integrated in a narrative context (throwing balls towards buoys floating in a pond).

This task [22] is a visual-spatial memory span task involving remembering a sequence of cubes pointed by the computer and reproducing the sequence in the same order by clicking with the mouse on screen elements. Thus, the cubes have been selected by a mouse click; in the following sections, we will use the term “clicked cubes” to designate this action. The material (Figure 1A) used is consistent with the study by Kessels et al [23] that proposed for each cube a number which is not visible by the participants (Figure 1B). Furthermore, 5 trials involving the memorization of 5 cubes (intermediate level as adopted from a study by Louis et al [21]) are proposed. The five sequences of cubes were (1) 5-2-1-8-6, (2) 4-2-7-3-1, (3) 3-9-2-4-8, (4) 7-8-2-9-4, and (5) 4-2-6-8-1 (Figure 1B).

The sequence of cubes for the training session was 2‐6.

Performance dimensions include clicked cubes (number of cubes that are clicked by the participant), correct cubes (the cubes correctly recalled whatever the order)*,* false cubes (the cubes incorrectly recalled)*,* omissions (failure to press a cube), total time, and expected responses (common performance indicator for all tasks) corresponds to this calculation: 100×(25–(False cubes+Omissions)÷25) and exact sequences (number of cubes clicked in the expected order and position) which can be compared with the terminology of memory or block span, corresponding to the length of the last correctly repeated sequence, in a traditional Corsi test [23]. However, our task did not aim to determine a memory span, as is typically the case in Corsi tests. In a memory span assessment, the goal is to identify the maximum number of items a participant can correctly retain and reproduce. In contrast, our task focused on exact sequences, and the instructions provided to participants did not explicitly emphasize memory span as a performance objective. Therefore, we have chosen to retain the term exact sequences to better reflect the specific nature and goals of our task.

#### Playful Corsi

This task is identical to the classic version, but cubes are replaced by 9 buoys with different colors integrated in a narrative context (throwing balls toward buoys floating in a pond; Figure 1C). This task uses the same performance metrics as Classic Corsi.

To verify hypothesis 2 (the multiplicity of cognitive solicitation might decrease PP, positive mental effort, and performances), we needed a task that solicited another cognitive function. We called it “Playful Corsi Multi.”

#### Playful Corsi Multi

Following several pretests in which various cognitive functions (such as mental flexibility or working memory updating) were compared, it was observed that mental motor inhibition, as required in tasks such as go no-go [24], was the most suitable cognitive function to integrate with the Corsi test. This choice minimized the impact on the extrinsic characteristics of the task while maintaining its core objectives.

**Figure 2.**
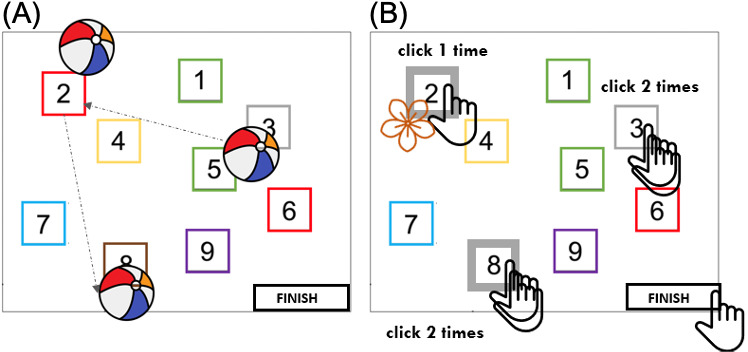
Schematic figures of the Playful Corsi Multi. (A) Ball displacement corresponding to memorization instructions given to participants; (B) actions required by the participants to respond to the instructions (2 clicks if there is no flower on the buoys and 1 click if there is a flower on the buoys).

The Playful Corsi Multi uses the same material as Playful Corsi (Figure 1). Participants memorize a sequence of 5 ball throws, reproducing it by clicking twice on buoys. During sequence reproduction, flowers may appear on buoys that must be clicked. In this case, participants do not have to make the second click on the gray buoy with a flower (no-go), and the response is validated with a single click (Figure 2). Furthermore, 4 pointing sequences with 2 no-go targets (gray buoy with flower) are proposed. In all sequences, a “trap” buoy with a flower on a nongray buoy is present and must be clicked twice, as similarly required for a buoy (of any color) without a flower. The sequence of 5 pointed buoys for the training session was with 1 no-go and 1 ‘’trap’’ buoy with a flower on a nongray buoy.

Performance dimensions encompass false buoys, false alarms (2 clicks instead of 1 upon the appearance of a no-go target), omissions (failure to perform a second click on a buoy), clicked buoys, exact buoys (number of buoys clicked in the expected order and position), expected responses (correspond to the accuracy and are calculated as 100×[25−(False alarms+omissions)÷25], *exact sequences* (number of buoys clicked in the expected order and position), reaction time for correct responses (the buoys correctly recalled), reaction time for all items combined, and total time (between the appearance of the red buzzer [finish button] and pressing it).

### Subjective Measures

Studies by Rubio et al [25] and Paxion [26] indicate that WP and NASA-TLX questionnaires complement each other. Furthermore, our study aimed to assess MWL related to attentional resources (WP questionnaire) and cognitive functions, emphasizing mental demands and mental effort (NASA-TLX questionnaire).

### NASA-TLX Questionnaire

NASA-TLX [2] evaluates perceived workload across 6 distinct subscales. In total, 3 dimensions relate to activity, that are mental demands (requirements for mental and perceptual activity)*,* physical demands (requirements for physical activity)*,* and temporal demands (sense of time pressure), 2 dimensions relate to strategies, that are performance (feeling of success in achieving the objectives set by the experimenter or oneself) and effort (the mental or physical effort required to reach the performance level targeted), and 1 dimension relate to emotional state, that is frustration (irritation, stress, insecurity, and discouragement). Following each task level, participant score each dimension from 0 (no demand) to 100 (maximum demand). In our study, we used the unweighted version of the questionnaire by calculating an average of the 6 dimensions to derive a raw task load index validated by Byers et al [27].

### WP Questionnaire

The WP questionnaire [4] asked the participants to provide the proportion of attentional resources across 8 subscales. Indeed, the dimensions of workload in this method were aligned with those in the multiple resource theory (described in Introduction). According to Tsang and Velazquez [4], 2 dimensions of WP questionnaire relate to “stages of processing”: perceptual or central processing (attentional resources required for activities like perceiving, remembering, problem-solving, and decision making) and response selection and execution (attentional resources required for response selection and execution; eg, the selection of the appropriate pedal to stop an automobile). Furthermore, 2 other dimensions are related to “processing codes”: spatial processing (according to Tsang and Velazquez [4], for tasks that are spatial in nature) and verbal processing (for tasks that are verbal in nature). In addition, 2 other dimensions are related to “input modality”: visual processing (for tasks that are performed based on the visual information received) and auditory processing (for tasks that are performed based on the auditory information received). And finally, 2 dimensions are related to “output modalities”: manual output (for tasks that required considerable attention for producing manual response), and speech output (for other tasks requiring considerable attention for producing speech responses).

Participants score each dimension from 0 (no demand) to 100 (maximum demand) representing the proportion of allocated attentional resources.

To reduce the time spent on questionnaires and given the absence of problem solving but also considering that the NASA-TLX’s mental demand dimension also questioned this source of MWL, the dimension perceptual or central processing was not considered. For the same reasons, because of the absence of actioners’ selection, auditory and speech tasks in our study, the dimensions response selection and execution, auditory processing, and speech output were not considered. Thus, only 4 of the 8 dimensions were evaluated. Indeed, as demonstrated by Tsang and Velazquez [4], the dimensions can be considered independently and rated in a 1D way. Therefore, we decided to focus on the spatial processing (WP3) and visual processing (WP5) as Corsi test is a visuo-spatial task, verbal processing (WP4) as the participants mentally verbalize the go no-go items (eg, the buoys colors), and manual output (WP7) dimensions, in relation to the participant’s movement of the mouse to click on the various items.

### PP Measure

Regarding PP, we assessed it using a question presented on a Likert scale ranging from 0 to 100: “To what extent did you find the task you have just completed playful and fun?”

### Experimental Procedure

After receiving approval from the Northwest III Ethics Committee for the Protection of Persons, the recruitment started. Potential participants received a study summary via email, followed by individual meetings held 7 days before the Nantes University Hospital study. Then, participants signed a consent letter. The experimental phase proceeded as follows: Participants were welcomed at Nantes University Hospital. Their eligibility and nonopposition to participation in the study were verified and they completed a pretest questionnaire (age, gender, and level of education). Then, they began the experiment. Each task had a training session (refer to Task and Procedure section) and after each task level, participants received NASA-TLX and WP questionnaires, and PP measure. Including questionnaires, each Corsi test (classic and playful one) has a total duration of 11 min and Playful Corsi Multi has a total duration of 16 min.

### Data Analysis

Raw data is available in Multimedia Appendix 2. Subjective measures (NASA-TLX, WP, and PP) were normalized through Cousineau correction [28]. This normalization allows for more accurate estimation of within‐participants CIs for subjective data in the case of experimental designs with several conditions. Cousineau correction was described as follows. Let *y_ij_* be the *i*th participant’s score in the *j*th condition (*i*=1, ..., *N; j*=1, …, M). Then, the normalized observations *z_ij_* were defined as follows:


$$z_{ij}=y_{ij}-\frac{1}{N}\sum_{j=1}^{M}y_{ij}+\frac{1}{NM}\sum_{i=1}^{N}\sum_{j=1}^{M}y_{ij}$$

Furthermore, some distributions of our data were not normal, and variances were not homogeneous. Thus, we used nonparametric statistics (Wilcoxon signed-rank tests and Conover post hoc comparisons) with the JASP (Jeffrey’s Amazing Statistics Program) software (version JASP 0.16; University of Amsterdam).

Finally, for the *P* value, the lower error threshold of .05 was considered significant.

## Results

### Comparisons Between Classic Corsi and Playful Corsi

#### Performance Measures

For the Corsi tests, 2 relevant performance dimensions appeared: expected responses and total time. The other Corsi performances (clicked cubes, correct cubes, false cubes, omissions, and exact sequences) were included within expected responses dimension (refer to Methods section).

**Figure 3.**
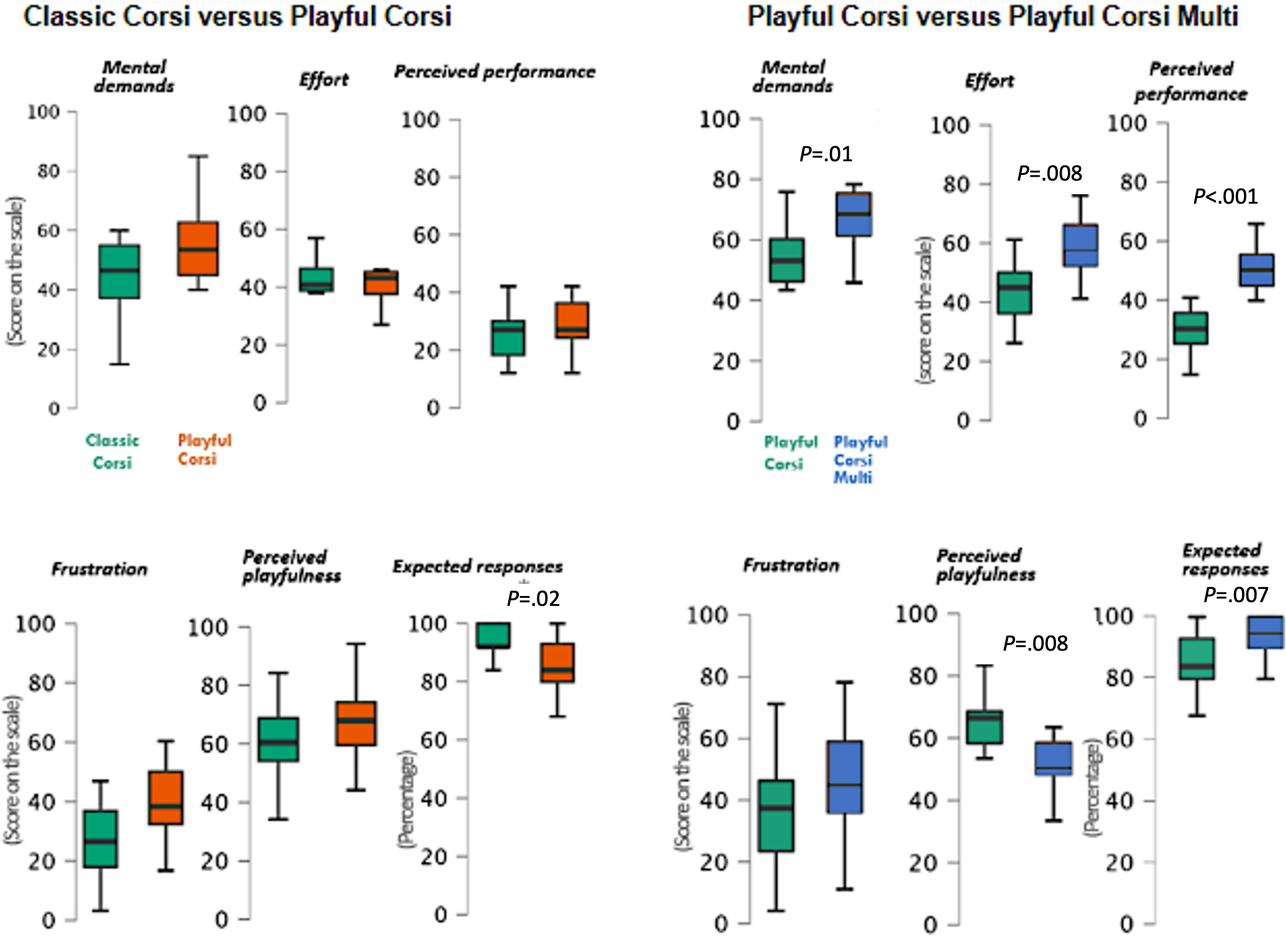
Comparisons of mental demands, effort, perceived performance, frustration, perceived playfulness, and expected responses between Classic Corsi and Playful Corsi and between Playful Corsi and Playful Corsi Multi.

Between the 2 tasks, expected responses and total time were significantly higher for the Classic Corsi than the Playful Corsi (Table 1; Figure 3).

**Table 1.** Statistical comparisons between Classic Corsi and Playful Corsi concerning performances data and mental workload questionnaires.

	Classic Corsi, mean (SD)	Playful Corsi, mean (SD)	W	*P* value
Expected responses	92.4 (9.1)	88.2 (11.3)	140.5	.02
Total time	10.7 (2.1)	6.8 (1.6)	209	<.001
Perceived playfulness	62.4 (8.8)	66 (8.8)	77	.30
Overall	187 (52.9)	186.3 (52.8)	65	.23
Mental demands	46.6 (13.8)	54.2 (13.9)	53	.16
Physical demands	13.6 (5.9)	13.4 (5.2)	36	.82
Temporal demands	35.6 (11.3)	38.9 (15.4)	93	.95
Effort	39.5 (11.7)	43.8 (13.6)	71	.54
Perceived performance	23.7 (10.4)	30.4 (9.9)	55.5	.19
Frustration	27.8 (16.8)	35.8 (16.8)	64.5	.23
WP3^a^	52.4 (12.1)	59.1 (12.1)	85.5	.48
WP5^b^	52.4 (10.9)	65.8 (10.9)	27.5	.04
WP7^c^	15.3 (12.8)	23.6 (12.8)	42.5	.30

a WP3: workload profile on spatial processing.

b WP5: workload profile on visual processing.

c WP7: workload profile on manual output.

#### NASA-TLX Questionnaire

Comparing Classic Corsi and Playful Corsi, the overall NASA-TLX score (corresponding to the sum of the 6 NASA-TLX dimensions which are on a scale of 600) showed no significant difference (Table 1), also for mental demands, physical demands, temporal demands, effort, performance, and frustration.

#### WP Questionnaire

We did not consider the WP4-verbal dimension since it was not a solicited dimension by the Corsi test, a visuo-spatial task (but it was solicited by Multi Playful Corsi test that contained mental inhibition based on the item colors; described in more detail in the Multicolored Playful Corsi vs Playful Corsi Multi section). Between the Classic Corsi and the Playful Corsi, only the WP5-visual dimension was significantly higher for the Playful Corsi than for the Classic Corsi (Table 1). But, between the 2 conditions, WP3-spatial and WP7-manual showed no significant difference (Table 1).

#### PP Measure

Between the 2 tasks, PP showed no significant difference (Table 1).

### Multicolored Playful Corsi vs Playful Corsi Multi

#### Performance Measures

Total time was not considered, as in the Playful Corsi Multi, there are 2 clicks per response, instead of 1 for the Multicolored Playful Corsi. Therefore, a trial-by-trial comparison was not possible. Concerning expected responses between the 2 tasks, they were significantly better for Playful Corsi Multi than Playful Corsi (Table 2; Figure 3).

**Table 2.** Statistical comparisons between Playful Corsi and Playful Corsi Multi concerning performances data and mental workload questionnaires.

	Playful Corsi, mean (SD)	Playful Corsi Multi, mean (SD)	W	*P* value
Expected responses	83.8 (13.9)	94 (5.5)	27	.007
Perceived playfulness	66 (11.9)	50.9 (11.9)	161	.008
Overall	216.6 (40.5)	284.4 (40.5)	25.5	.003
Mental demands	54.2 (9.4)	67.5 (9.4)	35.5	.01
Physical demands	13.4 (8.9)	15.4 (8.9)	33	.40
Temporal demands	38.9 (12.4)	46.6 (12.4)	56	.20
Effort	43.8 (9.9)	58.1 (9.9)	29	.008
Perceived performance	30.4 (7)	50.2 (7)	1	<.001
Frustration	35.8 (17.7)	46.5 (17.7)	63	.20
WP3^a^	59.1 (12.6)	64.4 (12.6)	81.5	.40
WP4^b^	10.2 (11.7)	16.3 (11.7)	14	.30
WP5^c^	65.8 (7.4)	68.3 (7.4)	71	.50
WP7^d^	23.6 (14.7)	21.1 (14.7)	93	.80

a WP3: workload profile on spatial processing.

b WP4: workload profile on verbal processing.

c WP5: workload profile on visual processing.

d WP7: workload profile on manual output.

#### NASA-TLX Questionnaire

The overall MWL was significantly higher for Playful Corsi Multi than Playful Corsi (Table 2; Figure 3), as were the mental demands, effort, and performance. However, physical demands, temporal demands, and frustration showed no significant difference between the 2 tasks.

#### WP Questionnaire

Between the tasks, the WP3, WP4, WP5, and WP7 showed no significant difference (Table 2; Figure 3).

#### PP Measure

PP was significantly higher for the Playful Corsi compared to the Playful Corsi Multi (Table 2; Figure 3).

## Discussion

### Principal Objectives

In this study we wanted to explore (1) how extrinsic variable (visual GLEs within a narrative context) impacts PP, positive mental effort, and performances at the very first stage of facing with a task devoted to cognitive training, and (2) if this effect endures while manipulating intrinsic variable (multiplicity of cognitive solicitation in a narrative context).

### Impact of Visual GLEs on PP and Positive Mental Effort

Our first hypothesis that visual GLEs integrated with a narrative context increase PP and positive mental effort is invalidated for various reasons. First, we observed no difference in terms of PP between Classic Corsi and its playful version. In the classic version of the Corsi, the PP is not poor but falls short of optimal. Consequently, we could have potentially improved the PP by manipulating the visual GLEs, but this was not the case. In this way, the PP did not seem to depend on the visual aspect of the task.

Concerning the supposed action of visual GLEs on positive mental effort, it appears that mental effort, perceived performance, and frustration did not differ between the 2 tasks. Thus, visual GLEs are not enough increasing positive mental effort.

On the contrary, MWL related to the mobilization of visual resources increased when we manipulated the extrinsic variables to task requiring (presence of visual GLEs). This is not surprising as, in the Playful Corsi, there are additional visual elements, such as waves or water lilies, which increase the MWL based on visual processing. Nevertheless, no other difference was noted about spatial processing and manual output, indicating that the tasks are relatively similar on the other dimensions.

Concerning the impact on effective performances, visual GLEs decreased the total time compared with a classic design, potentially attributed to the inherent challenge of game-like environments. There are no speed instructions for this task. Speed is therefore neither a performance criterion nor an objective to be achieved. The increase in speed observed in the playful condition may be due to an implicit incentive to accelerate, given the game-like design of the task, where speed is often an inherent goal. Concerning the expected responses (corresponded to the accuracy), our result contradicted the study by Redlinger et al [11] which specified that visual GLEs did not affect performance. Indeed, in the study by Redlinger et al [11], the presence of backgrounds led to slight reductions in accuracy but not significantly. In our study, this result is to consider in light to the observed impact on the response time. Indeed, we can assume that going faster leads to more errors. This can be linked to the well-known speed-accuracy trade-off [29], that is, the accuracy of a response varies with the time taken to produce it. Furthermore, given that in our study, the visual GLEs included changes to the background, additionally to the object on which participants had to interact (cubes or buoys), this could have induced greater distraction for participants and therefore significant differences in performance in favor of the Classic Corsi.

In conclusion, our results were in line with the meta-analysis of Vermeir et al [9], indicating that gamified tasks are perceived as significantly more demanding, at least considering the perceived demand. Furthermore, this gamification is not accompanied with a voluntary mental effort improvement nor a better PP. A deterioration in performance has even been observed. So, adding visual GLEs with narrative context is not always beneficial for the task.

### Impact of Increasing Cognitive Demanding

Our second hypothesis that the multiplicity of cognitive demanding of the playful task might decrease PP and positive mental effort is partially confirmed. PP is negatively influenced by intrinsic variables (solicitation of multiple cognitive functions) but there is every reason to believe that this manipulation has increased the positive mental effort. This is supported by a higher perceived mental effort in conjunction with a higher perceived performance (confirmed by better effective performances) but no increase in the perceived frustration.

Concerning the decrease in PP, increasing the solicitation of cognitive processing increases mental demand, which could have a negative impact on PP. Indeed, Fang et al [17] and Donovan et al [30] showed that PP had a significant and negative correlation with task complexity. In our study, this could also stem from the way we question the notion of PP: “To what extent did you find the task you have just completed playful and fun?” As the task becomes more cognitively demanding, it can be perceived as more “serious,” which could reduce the feeling of “playful.” Also, we acknowledge the limitation of combining 2 descriptors (“playful” and “fun”) in a single question, as they may not hold the same meaning for all participants. “Playful” could refer to the task’s design or its gamified elements, while “fun” might reflect the participant’s subjective enjoyment or emotional response. This overlap introduces potential ambiguity, as participants may weigh one descriptor more heavily than the other. For the next studies, we will consider separating these descriptors into distinct questions to capture more nuanced insights into PP and enjoyment, thereby reducing interpretive variability.

Nevertheless, even though the augmentation of the complexity of a cognitive task decreases the PP, this manipulation increases mental effort and perceived performance without increasing frustration. This is the opposite of what we expected to see. This can stem from the fact that “serious” is not antagonistic to “pleasant,” “enjoyable,” or “motivating.” It just means that what would help induce greater voluntary mental effort, would correspond more to stronger cognitive stimulation (agreed) than just the “fun” characteristics of an activity. This means that, to encourage the voluntary mobilization of mental resources, one possibility is to manipulate the cognitive demanding of a task rather than its design only. Here, by “cognitive demanding,” we are talking about cognitive challenge, not difficulty. In fact, the actual performance, which is better in this condition, shows us that the task is not more complex to perform. But everything seems to indicate that the participant is more determined to perform. Such a performance boost, resulting from cognitive function multiplicity, could be a drawback when performing cognitive assessment but is likely to be desirable in a cognitive training scenario [14]. Indeed, in cognitive training scenarios, the goal is to enhance overall cognitive functioning by engaging multiple cognitive processes simultaneously since it mimics the complexity and demands of real-life situations where multiple cognitive functions are often required at once.

Therefore, to encourage the voluntary mobilization of mental resources, based on our results, a possible approach could be to manipulate the cognitive demanding of a cognitive task (increasing mental effort and performance without increasing frustration) rather than its design but allowing it to maintain a high level of performance.

### Limitations

Finally, it is important to specify that this study should be extended to include a larger number of participants to strengthen statistical significance. Given that the experiments were carried out at the Nantes Hospital, which is far from the city center and has few medical staff available, we were constrained by logistical considerations. In addition, the choice of terms used for measuring PP was not without ambiguity and may require refinement in future studies.

### Conclusion and Implications for Research

This study can provide cognitive testing practitioners or even social game practitioners with valuable information on specific game-like guidelines to impact users’ PP, while taking into consideration the impact on MWL. We showed that the PP is a skillful balance between design and the solicitation of cognitive functions. Possibly, visual GLEs could raise the stakes of the task slightly and implicitly encourage people to go a bit faster (as observed with the Corsi test), but working on the way cognitive functions are solicited would be wiser to improve users’ voluntary mental effort than adding GLEs. Future research could explore further this last hypothesis as well as the influence of additional GLEs, such as sounds, variations in narrative context, or performance feedback.

## Supplementary material

10.2196/63491 Multimedia Appendix 1 Video demonstration of the tasks used in the study.

10.2196/63491 Multimedia Appendix 2 Raw data on how to encourage the voluntary mobilization of mental resources in a cognitive task.

## Abbreviations

GLE: game-like element
JASP: Jeffrey’s Amazing Statistics Program
MWL: mental workload
NASA-TLX: NASA-Task Load Index
PP: perceived playfulness
WP: workload profile
WP3: workload profile on spatial processing
WP4: workload profile on verbal processing
WP5: workload profile on visual processing
WP7: workload profile on manual output
